# Handling an uncertain control group event risk in non-inferiority trials: non-inferiority frontiers and the power-stabilising transformation

**DOI:** 10.1186/s13063-020-4070-4

**Published:** 2020-02-06

**Authors:** Matteo Quartagno, A. Sarah Walker, Abdel G. Babiker, Rebecca M. Turner, Mahesh K. B. Parmar, Andrew Copas, Ian R. White

**Affiliations:** MRC Clinical Trials Unit, Institute of Clinical Trials and Methodology, University College London, 90 High Holborn, Second Floor, London, WC1V 6LJ UK

**Keywords:** Non-inferiority, Resilience, Power-stabilising transformation

## Abstract

**Background:**

Non-inferiority trials are increasingly used to evaluate new treatments that are expected to have secondary advantages over standard of care, but similar efficacy on the primary outcome. When designing a non-inferiority trial with a binary primary outcome, the choice of effect measure for the non-inferiority margin (e.g. risk ratio or risk difference) has an important effect on sample size calculations; furthermore, if the control event risk observed is markedly different from that assumed, the trial can quickly lose power or the results become difficult to interpret.

**Methods:**

We propose a new way of designing non-inferiority trials to overcome the issues raised by unexpected control event risks. Our proposal involves using clinical judgement to specify a ‘non-inferiority frontier’, i.e. a curve defining the most appropriate non-inferiority margin for each possible value of control event risk. Existing trials implicitly use frontiers defined by a fixed risk ratio or a fixed risk difference. We discuss their limitations and propose a fixed arcsine difference frontier, using the power-stabilising transformation for binary outcomes, which may better represent clinical judgement. We propose and compare three ways of designing a trial using this frontier: testing and reporting on the arcsine scale; testing on the arcsine scale but reporting on the risk difference or risk ratio scale; and modifying the margin on the risk difference or risk ratio scale after observing the control event risk according to the power-stabilising frontier.

**Results:**

Testing and reporting on the arcsine scale leads to results which are challenging to interpret clinically. For small values of control event risk, testing on the arcsine scale and reporting results on the risk difference scale produces confidence intervals at a higher level than the nominal one or non-inferiority margins that are slightly smaller than those back-calculated from the power-stabilising frontier alone. However, working on the arcsine scale generally requires a larger sample size compared to the risk difference scale. Therefore, working on the risk difference scale, modifying the margin after observing the control event risk, might be preferable, as it requires a smaller sample size. However, this approach tends to slightly inflate type I error rate; a solution is to use a slightly lower significance level for testing, although this modestly reduces power. When working on the risk ratio scale instead, the same approach based on the modification of the margin leads to power levels above the nominal one, maintaining type I error under control.

**Conclusions:**

Our proposed methods of designing non-inferiority trials using power-stabilising non-inferiority frontiers make trial design more resilient to unexpected values of the control event risk, at the only cost of requiring somewhat larger sample sizes when the goal is to report results on the risk difference scale.

## Introduction

Often a new treatment is expected not to have greater efficacy than the standard treatment, but to provide advantages in terms of costs, side-effects or acceptability. Here, a non-inferiority trial [1] can test whether the new treatment’s efficacy is not unacceptably lower than standard treatment, and also, where relevant, guarantee that a minimum acceptable treatment effect relative to a hypothetical placebo is preserved, while providing sufficient evidence of superiority on secondary outcomes to support its use. Non-inferiority designs have been increasingly used in recent years [2].

A critical design choice is the non-inferiority margin, which is the largest acceptable loss of efficacy [3]. Considerations regarding margin choice depend on the type of primary outcome. We focus here on binary outcomes, for which either absolute [4] (risk difference) or relative [5] (risk ratio) margins can be defined. For example, the Food and Drug Administration guidelines [6] suggest that for licensing trials, the results from placebo-controlled trials evaluating the standard treatment might directly inform margin choice, using the lower bound of the confidence interval for the estimated effect versus placebo, most often using the absolute scale. The largest tolerable effect size (e.g. risk difference or risk ratio) for the new treatment chosen with this strategy is referred to as M_1_. More commonly, the goal might be to preserve a certain proportion of the effect of the standard relative to placebo, which can be formulated as either an absolute or relative margin. In this case, we refer to the maximum tolerable effect size as M_2_ (where M_2_ = x% of M1). Using historical data to define M_1_ and M_2_ is often referred to as the ‘fixed-margin approach’ [7]. An alternative to defining a margin is the so-called ‘synthesis method’, which defines non-inferiority simply as preservation of the fraction x% of the standard effect relative to placebo [8]. In non-regulatory non-inferiority trials with a public health perspective, the margin is instead chosen to reflect clinical judgement on the value of the new treatment’s secondary advantages [9].

The choice between a relative or absolute margin depends on both clinical and statistical considerations; both the choice of scale and how to define margins have been discussed widely in the literature [3, 6, 8, 10–13] and we do not address these here. Clinically, a relative difference has the advantage of being potentially transferable to secondary outcomes. Statistically, though, it requires a much larger sample size.

In both cases, the expected control arm (standard treatment) event risk plays a very important role in the choice of the non-inferiority margin [12]. However, at trial completion, the actual control event risk can differ considerably from the expected one. This, which is sometimes referred to as a failure of the ‘constancy’ assumption between control event risks in the current trial and the previous placebo-controlled trials, can occur when prior information was not correct, for example when standard of care has improved over years [14], because a slightly different sub-population was recruited [4] or because additional aspects of care (or a Hawthorne effect) influenced outcomes in the control group. This can have serious consequences on the power, and hence the interpretation, of the trial, particularly when the expected control event risk is very large (e.g. > 90%) or small (< 10%): the latter is common in non-inferiority trials where existing treatments are often highly effective, precluding demonstrating superiority of a new treatment on the primary endpoint.

For example, for control risk < 50%, the sample size needed to achieve 90% power under a 5% non-inferiority margin on the risk difference scale (one-sided alpha = 2.5%) increases with the control event risk (Figure S1 in Additional file 1); hence, if the control event risk is larger than anticipated, this reduces the power of the trial to demonstrate non-inferiority (Figure S2 in Additional file 1). The opposite occurs when working on the risk ratio scale, so that a lower than expected control event risk reduces power. The difference arises because the variance of the risk difference increases as the risk increases towards 0.5, while the variance of the risk ratio decreases. We discuss a specific example illustrating this below (the OVIVA trial [15]). Furthermore, higher power than designed may not actually aid interpretation. For example, Mauri and D’Agostino [13] discuss the ISAR-safe [16] non-inferiority trial, where the observed control event risk was much lower than originally expected. The results provided strong evidence of non-inferiority based on the prespecified non-inferiority margin as a risk difference, but they were also consistent with a threefold increase in risk based on the risk ratio, and so the authors did not conclude non-inferiority.

A few solutions have previously been proposed to tackle lack of constancy in the analysis. For example, Koopmeiners and Hobbs [17] proposed a way to use Bayesian modelling to adapt the non-inferiority margin including historical data together with data from the current. Nie and Soon [18, 19] and Hanscom et al. [20] instead used observed data from the trial to establish whether the constancy assumption holds or whether the margin has to be modified using adjustment for baseline or post-randomisation covariates in the current trial.

Here we propose a different approach to non-inferiority trials, which protects against a lower or higher than expected control event risk, preserving power and interpretability of results. Our method can be prespecified at the trial design stage; under the public health perspective it is applicable when there are no previous placebo-controlled trials and no clear predictors of control event risk available. It allows a larger role for clinical judgement in determining whether and how the non-inferiority margin should depend on the control event risk.

## The non-inferiority frontier

Assume we want to test whether a new treatment T_1_ is non-inferior to the standard treatment T_0_. The primary (binary) outcome is an unfavourable event, e.g. death or relapse within one year from randomisation. Let:

- *π*_1_, *π*_0_ be the true incidences in the experimental and control groups, respectively;
- *π*_*e*1_, *π*_*e*0_ be the expected incidences assumed in the sample size calculation. Usually *π*_*e*1_ = *π*_*e*0_ but occasionally [4] studies are designed with *π*_*e*1_ < *π*_*e*0_ or *π*_*e*1_ > *π*_*e*0_;
- *π*_*f*1_ be the largest acceptable incidence in the experimental group if the control group incidence is *π*_*e*0_. In a trial with an unfavourable outcome, *π*_*f*1_ > *π*_*e*0_;
- *δ* be the non-inferiority margin, defined as *δ* = *π*_*f*1_ − *π*_*e*0_ if the risk difference scale is used and *δ* = log(*π*_*f*1_/*π*_*e*0_) if the (log-)risk ratio scale is used;
- *n*_1_, *n*_0_ be the sample sizes, with allocation ratio *r* = *n*_1_/*n*_0_.

Several recommendations have been given regarding choice of the most appropriate non-inferiority margin [3, 6], involving both clinical and statistical considerations. While sample size calculations allow for stochastic variation between the true control event risk *π*_0_ and its final observed estimate ${\hat{\pi}}_0$, they do not allow for substantial misjudgement in the envisaged truth. We therefore argue that it is insufficient to define non-inferiority in terms of a single margin *δ*; it is instead preferable, at the design stage, to define a curve associating a specific margin ${\delta}_{\pi_0}$ to each possible value of control event risk *π*_0_. We call this the non-inferiority frontier. The non-inferiority frontier describes our judgement if we knew the true values of *π*_0_ and *π*_1_; we discuss statistical inference from observed data in the ‘Implementation’ section.

### Risk difference versus risk ratio

**Fig. 1:**
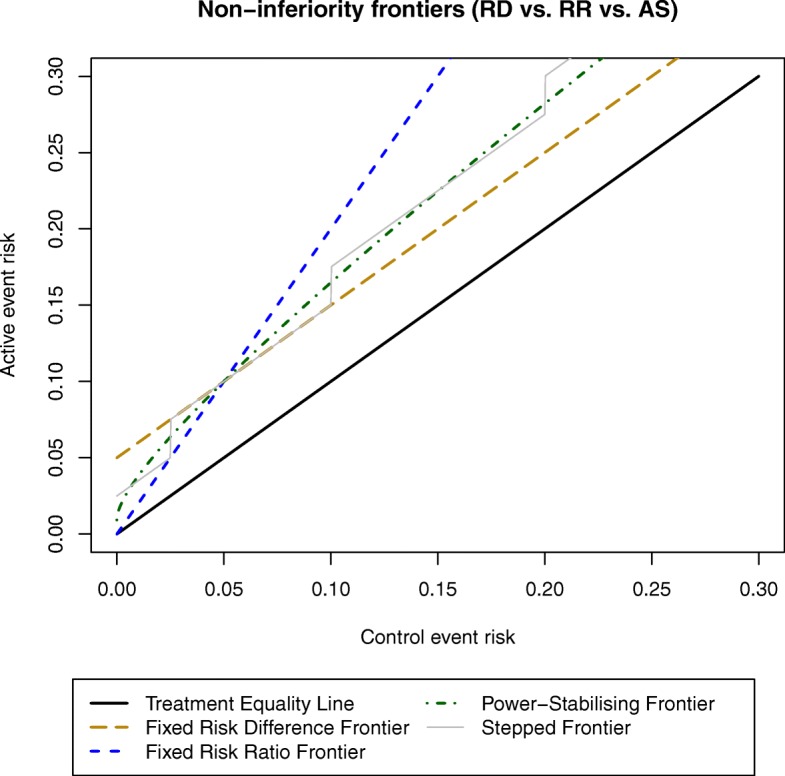
Non-inferiority frontiers: comparison of fixed risk ratio (2), fixed risk difference (5%) and power-stabilising frontiers. The black solid line corresponds to strict equivalence of the two treatments

The standard design, assuming a single non-inferiority margin *δ* irrespective of *π*_0_, corresponds to a fixed risk difference or fixed risk ratio frontier. These frontiers are shown in Fig. 1. The region underneath the golden line is the non-inferiority region assuming a fixed risk difference frontier; whatever the control event risk, the new treatment is non-inferior if *π*_1_ − *π*_0_ < 0.05. Similarly, the region below the blue line is the non-inferiority region assuming a constant risk ratio frontier.

The choice of frontier is important even when the expected control event risk is correct, i.e. *π*_*e*0_ = *π*_0_. As shown by Figs. S1 and S2 in Additional file 1, power and sample size calculations using different analysis scales give very different answers even when the assumed *π*_*f*1_ and *π*_*e*0_ are the same.

### Stepped frontiers

Another possible approach is to manually define the non-inferiority frontier choosing the non-inferiority margin for a range of plausible values of the control event risk, basing the choice on appropriate clinical considerations. Ideally the frontier would be a continuous smooth curve based on considering a very large number of values for the control event risk. In practice, though, clinical judgement is likely to be sought regarding the non-inferiority margin for a limited range of intervals in the control event risk, which leads to a step function similar to the grey solid line (based on a risk difference analysis scale) in Fig. 1.

### The power-stabilising non-inferiority frontier

We propose a further choice of frontier, the fixed arcsine difference [21, 22] frontier, i.e. constant $\mathrm{asin}\left(\sqrt{\pi_{f1}}\right)-\mathrm{asin}\left(\sqrt{\pi_{e0}}\right)$. Although the arcsine difference is more difficult to interpret than other measures, it generally represents an intermediary between the fixed risk difference and risk ratio frontiers and might thus be very close to a continuous frontier based on clinical opinion (see discussion of OVIVA below). Furthermore, its major advantage is that its asymptotic variance is independent of *π*_0_. Hence, when using a fixed arcsine difference frontier, the sample size and power calculations are approximately unaffected by *π*_*e*0_ − *π*_0_. We therefore call this the power-stabilising non-inferiority frontier, represented by the dark green line in Fig. 1.

### Choosing the non-inferiority frontier

The most appropriate non-inferiority frontier must be chosen using clinical, as well as statistical, arguments.

#### Clinical considerations

If the investigators’ only interest lies in the single binary efficacy outcome, an increase in event risk from 5% to 10% can be considered as undesirable as an increase from 45% to 50%; in both, the experimental treatment leads to 50 more events per 1000 patients and a fixed risk difference frontier might be appropriate. However, many investigators would feel that the former increase is more important than the latter. This could be justified by arguing that a relative effect measure is more likely to be transportable to other outcomes or more closely matches opinions of clinicians or patients. In this case, as the control event risk increases, we might tolerate a larger absolute increase in intervention event risk. However, as shown in Fig. 1, with the risk ratio frontier, the maximum tolerable absolute difference quickly becomes very large as the control event risk increases beyond that originally anticipated. A clinically determined frontier is theoretically appealing, but drawing such a frontier in practice is challenging; the only simple option is a step function as shown in Fig. 1, but under this frontier the margin for very similar control risks could be quite different; for example, the margin selected for an observed control event risk ${\hat{\pi}}_0=9.9\%$ in Fig. 1 would be 2.5% different from that for ${\hat{\pi}}_0=10\%$. A continuous function would be preferable, but it is not clear how such a curve could be derived. The power-stabilising frontier is a good compromise between the risk ratio and risk difference frontiers. Because of this, although it does not directly come from clinical considerations, it often returns values that are very close to those that researchers would choose for the clinically determined frontier.

As an example, the OVIVA [15] trial aimed to determine whether oral antibiotics were non-inferior to intravenous antibiotics to cure bone and joint infections. Intravenous antibiotics were the standard based on historical precedent, not evidence. Based on pilot data from one tertiary referral centre, researchers expected a low control event risk of treatment failure (*π*_*e*0_ = 5%); given this, they were happy to tolerate up to a 10% event risk for the experimental treatment, because of its substantial advantages (e.g reduced line complications, earlier hospital discharge), i.e. a 5% absolute margin. However, the observed pooled event risk across 29 centres of varying sizes was much higher $\left({\hat{\pi}}_0=12.5\%\right)$; assuming this reflected the control group risk, they were happy to tolerate an experimental event risk larger than implied by the same fixed risk difference frontier (*π*_*f*1_ = 17.5%). As the risk ratio increases with control risk, a fixed risk ratio frontier (*π*_*f*1_ = 25%) was an alternative in this case. However, the investigators decided that the maximum tolerable experimental event risk given *π*_0_ = 12.5% was *π*_*f*1_ = 20%, which is very close to the arcsine frontier (*π*_*f*1_ = 19.5%).

#### Statistical considerations

Designing and analysing a trial using a fixed risk difference or risk ratio frontier is the same as designing and analysing a standard non-inferiority trial, with the non-inferiority margin held fixed. Keeping the same fixed risk difference or fixed ratio frontier, regardless of the final control event risk, is what is currently done in most trials, although usually there is no prespecified frontier, and if the observed control group (or pooled) event rate is observed to differ markedly from that anticipated, researchers may decide to change the margin to something else considered more appropriate margin, as in OVIVA. However, this strategy is prone to inflation of type 1 error, as it uses the data to inform the margin. Therefore, this approach should only be used combined with some method for controlling type 1 error, for example inflating standard errors or using a lower significance level α.

The power-stabilising frontier could be easily implemented by designing and analysing a trial using an arc-sine difference margin, but results would be difficult to interpret clinically. We discuss alternative ways of implementing the power-stabilising frontier in the next section.

Another aspect to consider when choosing the frontier is that sample size calculations give very different answers when working on different scales. In an example trial with one-sided α = 2.5%, power = 90%, *π*_*e*0_ = 5%, and *π*_*f*1_ = 10%, the sample size to show non-inferiority on the arcsine scale (568 patients/group) is larger than on the risk difference scale (400 patients/group; 5% absolute margin); hence, choosing the arcsine frontier may require up to 40% more patients. However, the sample size required to show non-inferiority on the risk ratio scale is larger still (832 patients/group; twofold relative risk margin).

## Implementation

There are several ways we could design and analyse a trial under the power-stabilising frontier. We introduce them here and provide an illustrative analysis example in Additional file 1.

### Test and report on the arcsine scale

The simplest solution is to design the trial prespecifying the non-inferiority margin on the arcsine difference scale; it is then sufficient to test non-inferiority at this fixed margin and report a point estimate and confidence interval on the arcsine scale, regardless of the final observed control event risk. However, such results are not easily interpretable and are unlikely to be clinically acceptable.

### Test on the arcsine scale, report on the risk difference scale

A second possibility is to design the trial and perform the test on the arcsine scale, but report results on the risk difference (or risk ratio) scale. The problem here is that the test statistic may not correspond to the relationship of the margin to the confidence interval. We propose two ways to resolve this; we present them for the risk difference scale, although they could be easily adapted to the risk ratio scale. Given an estimated arcsine difference $\hat{AS}$ with associated standard error ${\hat{\sigma}}_{AS}$, a fixed non-inferiority margin on the arcsine difference scale *δ*_*AS*_ and an estimated risk difference $\hat{RD}$ with standard error ${\hat{\sigma}}_{RD}$:

#### Back calculation of margin

1. Calculate the Z statistic for the arcsine scale test:

$$ {Z}_{AS}=\frac{\hat{AS}-{\delta}_{AS}}{{\hat{\sigma}}_{AS}} $$

2) Calculate for what non-inferiority margin *δ*_*RD*_ we get the same Z statistic when testing on the risk difference scale:

$$ {\delta}_{RD}=\hat{RD}-{Z}_{AS}\cdotp {\hat{\sigma}}_{RD} $$

3) Report the confidence interval on the risk difference scale and *p* value of the test for non-inferiority at margin *δ*_*RD*_:
  $$ p={\varPhi}^{-1}\left({Z}_{AS}\right)\kern1.25em CI\left(1-\alpha \right)=\left(\hat{RD}-{z}_{1-\alpha}\cdotp {\hat{\sigma}}_{RD};\hat{RD}+{z}_{1-\alpha}\cdotp {\hat{\sigma}}_{RD.}\right) $$

#### Back calculation of significance level and modification of margin

1. Calculate the non-inferiority margin ${\delta}_{RD}^{\ast }$ on the risk difference scale corresponding to *δ*_*AS*_ on the arcsine scale for the observed value of control risk ${\hat{\pi}}_0$:

$$ {\delta}_{RD}^{\ast }=\mathit{\sin}{\left( asin\left(\sqrt{{\hat{\pi}}_0}\right)+ asin\left(\sqrt{\pi_{f1}}\right)- asin\left(\sqrt{\pi_{e0}}\right)\right)}^2-{\hat{\pi}}_0 $$

2) Calculate the Z statistic *Z*_*RD*_ for the test on the risk difference scale:

$$ {Z}_{RD}=\frac{\hat{RD}-{\delta}_{RD}^{\ast }}{{\hat{\sigma}}_{RD}} $$

3) Calculate at what significance level α^∗^ the test using *Z*_*RD*_ would be equivalent to a α-level test using *Z*_*AS*_:

$$ {z}_{1-{\alpha}^{\ast }}={z}_{1-\alpha}\frac{Z_{RD}}{Z_{AS}} $$

4) Report (1 − α^∗^) confidence interval on the risk difference scale and *p* value of the test for non-inferiority at margin ${\delta}_{RD}^{\ast }$:
  $$ p={\varPhi}^{-1}\left({Z}_{AS}\right)\kern1.25em CI\left(1-{\upalpha}^{\ast}\right)=\left(\hat{RD}-{z}_{\left(1-{\upalpha}^{\ast}\right)}\cdotp {\hat{\sigma}}_{RD};\hat{RD}+{z}_{\left(1-{\upalpha}^{\ast}\right)}\cdotp {\hat{\sigma}}_{RD}\right) $$

Both approaches are potentially valid; when *π*_0_ < 50%, the adjustment is generally small and, most notably, confidence levels reported are larger than the nominal (1 − α). One difficulty with this approach is that the sample size might be impractically large for a design based on the arc-sine scale, particularly for small values of control event risk (where the frontier tends to the same value, Fig. 1), if the ultimate goal is to report on the risk difference scale, for the reasons discussed in Section 2.4. Conversely, since sample size required to demonstrate non-inferiority on the risk ratio scale is larger than on the arcsine scale, the non-inferiority margin *δ*_*RR*_ or the significance level α^∗^ may be unacceptably large when the goal is to report on the risk ratio scale.

### ‘Conditionally modify margin’: modify non-inferiority margin after observing control group event risk

Our favoured proposal is to design the trial using a standard risk difference or risk ratio margin *δ* and then modify the margin to *δ*^∗^ only if the observed event risk ${\hat{\pi}}_0$ differs by more than a certain threshold *ϵ* from the expected *π*_*e*0_. Specifically:

- At trial completion we observe ${\hat{\pi}}_0$;
- If $\left|{\hat{\pi}}_0-{\pi}_{e0}\right|>\epsilon$ (risk difference scale) or $\left|\log \left({\hat{\pi}}_0/{\pi}_{e0}\right)\right|>\epsilon$ (risk ratio scale), then:
  - ◦ Find ${\pi}_{f1}^{\ast }$ that solves $\mathrm{asin}\left(\sqrt{\pi_{f1}^{\ast }}\right)-\mathrm{asin}\left(\sqrt{{\hat{\pi}}_0}\right)=\mathrm{asin}\left(\sqrt{\pi_{f1}}\right)-\mathrm{asin}\left(\sqrt{\pi_{e0}}\right)$;
  - ◦ Modify non-inferiority margin to ${\delta}^{\ast }={\pi}_{f1}^{\ast }-{\hat{\pi}}_0$ (risk difference) or ${\delta}^{\ast }=\log \left(\frac{\pi_{f1}^{\ast }}{{\hat{\pi}}_0}\right)$ (risk ratio);
  - ◦ Test non-inferiority at margin *δ*^∗^;
- Otherwise do not modify margin and test non-inferiority at *δ*.

This approach, while preserving the simplicity in interpreting non-inferiority against risk differences or risk ratios, potentially helps preserve power and interpretability when the true control event risk is badly misjudged by modifying *δ* according to the power-stabilising frontier. Differently from the method in Section 3.2(ii), the margin is only modified when the observed control risk differs substantially from its expectation. However, since the margin is modified in a data-dependent way, the method is potentially prone to inflation of type I error. We explore this next.

### Type I error and power of the ‘conditionally modify margin’ method

We simulate 100,000 datasets for a range of designs and true incidences, starting from a base-case scenario and then investigating alternatives, changing simulation parameters one-by-one (Table 1), appropriately calculating sample size from the design parameters in Table 1 and the formulae in the additional material. Since sample size calculations give very different answers when using risk ratio or risk difference; we generate different datasets for the two effect measures.

**Table 1:** Design parameters of the different simulation scenarios. ***π***_***e*0**_ and ***π***_***e*1**_ represent the expected control and active event risk, ***π***_***f*1**_ the maximum tolerable active event risk and r the allocation ratio

Scenario	*π*_*e*0_ (%)	*π*_*e*1_	*π*_*f*1_ (%)	*r*	Power (%)
Base-case	5	=*π*_*e*0_	10	1	90
Alternative 1	**10**	=*π*_*e*0_	**15**	1	90
Alternative 2	5	$=\frac{{\boldsymbol{\pi}}_{\boldsymbol{e}\mathbf{0}}}{\mathbf{2}}$	10	1	90
Alternative 3	5	=*π*_*e*0_	**7.5**	1	90
Alternative 4	5	=*π*_*e*0_	**15**	1	90
Alternative 5	5	=*π*_*e*0_	10	**0.5**	90
Alternative 6	5	=*π*_*e*0_	10	**2**	90
Alternative 7	5	=*π*_*e*0_	10	1	**80**

In bold, design parameters that differ from the base-case scenario

#### Type I error

We consider 40 data-generating mechanisms for each scenario, with *π*_0_ in the range of 0.5%–20% and *π*_1_ derived under the non-inferiority null from the arcsine rule: $\mathrm{asin}\left(\sqrt{\pi_1}\right)-\mathrm{asin}\left(\sqrt{\pi_0}\right)=\mathrm{asin}\left(\sqrt{\pi_{f1}}\right)-\mathrm{asin}\left(\sqrt{\pi_{e0}}\right)$.

This is the appropriate data-generating mechanism for evaluating type I error assuming the power-stabilising frontier holds. We compare four different analysis methods:

1. Do not modify margin: simply test non-inferiority with margin *δ* on the risk difference/ratio scale;
2. Modify margin, with *ϵ*= 5% for risk difference or log(2) for log risk ratio;
3. Modify margin, with *ϵ*= 2.5% for risk difference or log(1.5) for log risk ratio;
4. Modify margin, with *ϵ*= 1.25% for risk difference or log(1.25) for log risk ratio.

##### Base-case

**Fig. 2:**
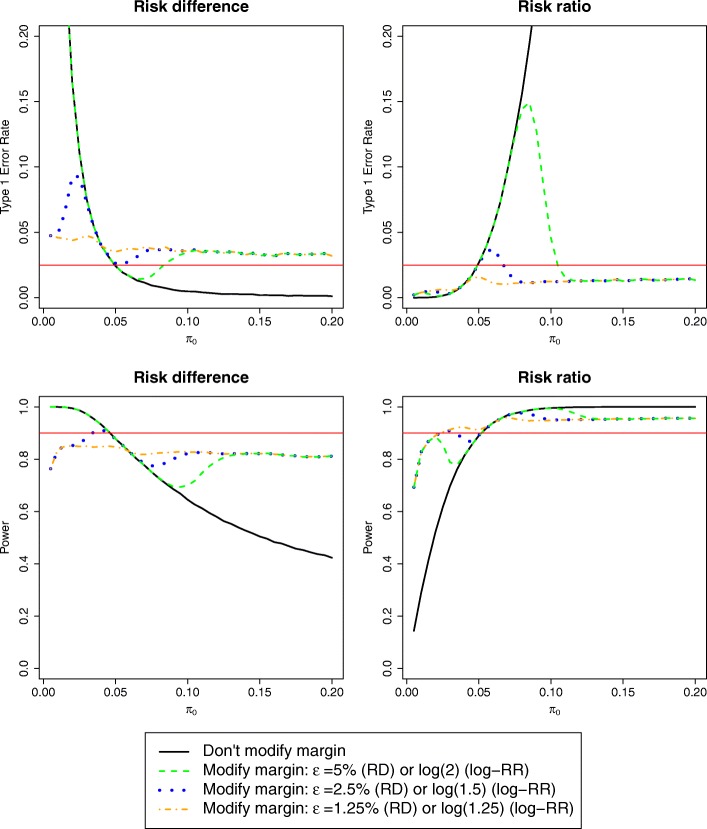
Type I error (top) and power (bottom) of ‘do not modify margin’ and ‘modify margin’ procedures, using the risk difference (left) or risk ratio (right) scale. Data are generated according to the base-case scenario of Table 1 for varying values of control event risk

Figure 2 shows the results of these simulations, designing and analysing the data on a risk difference (left) or risk ratio (right) scale. Given our chosen non-inferiority frontier, ‘do not modify margin’ leads to inflated type I error rate if the control event risk is lower or higher than expected using the risk difference or risk ratio respectively. The three ‘conditionally modify margin’ procedures are identical to ‘do not modify margin’ in a small region around the expected control event risk; the width of this region is directly proportional to the magnitude of *ϵ*. For *π*_0_ > 10%, the margin is almost always modified (Figure S3 in Additional file 1) and the ‘conditionally modify margin’ procedures have the same level of type I error. Using the risk ratio, this level is below the nominal 2.5%, while with the risk difference it is just above 3.5% for *π*_0_ > 5%.

Comparing the strategies with different *ϵ*, the procedure using the smallest threshold seems preferable irrespective of the scale used. In particular, when using risk ratios, it leads to a type I error always below 2.5%, while with risk difference the rate remains slightly inflated, to a maximum of 4%–5% at low event risks < 4%.

##### Other data-generating mechanisms

**Fig. 3:**
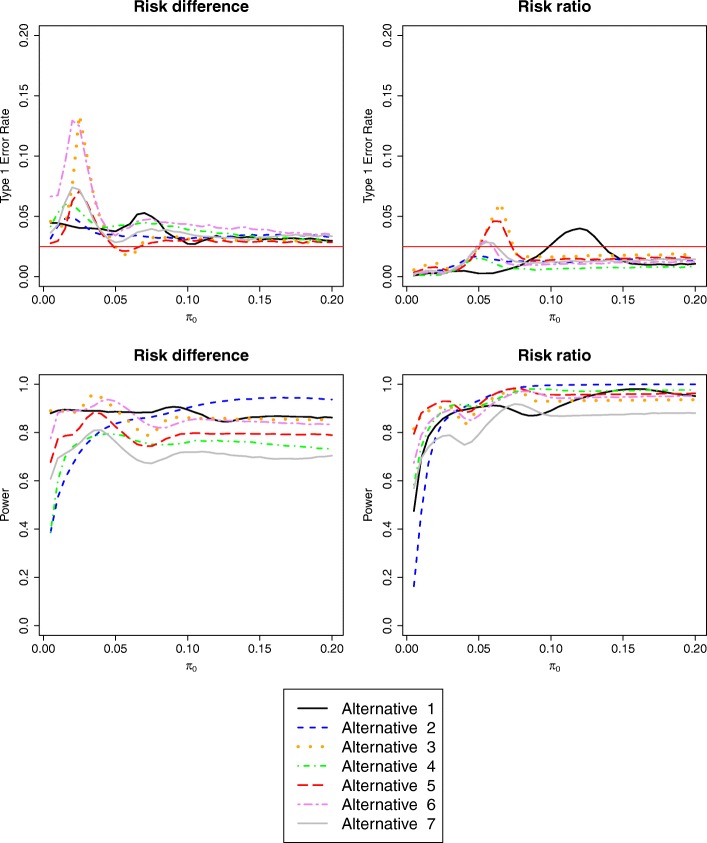
Type I error (top) and power (bottom) of the ‘conditionally modify margin’ procedure, using the risk difference (left) or risk ratio (right) scale. Data are generated according to the alternative scenarios of Table 1 for varying values of control event risk

Figure 3 shows the results for the alternative scenarios, using procedure 4 only, i.e. ‘conditionally modify margin’ with the smallest threshold (other procedures in Figs. S4 and S5 in Additional file 1). Allocation ratio (alternatives 5 and 6) has a greater impact than other factors, because with more patients allocated to control, the estimated risk is affected by less error. However, in general, conclusions are not altered substantially.

#### Power

We again vary *π*_0_ between 0.5% and 20%, but this time under the non-inferiority alternative with *π*_1_ = *π*_0_.

##### Base-case

Under ‘do not modify margin’, power is substantially reduced if *π*_0_ is higher (risk difference) or lower (risk ratio) than expected (Fig. 2). Using a risk ratio, the power of any of the ‘conditionally modify margin’ methods is always either above the nominal 90% or above the power of the ‘do not modify margin’ procedure. This also holds for the risk difference, except when *π*_0_ is lower than expected; nevertheless, power remains close to 80% even in this scenario. Interestingly, the procedure with the smallest threshold is the only one not achieving the nominal power when the control event risk is correct, possibly because the margin is at times modified even when risk differs from the expected only because of random variation.

##### Alternatives

Figure 3 shows the results under the alternative scenarios using procedure 4. The greatest difference from the base-case scenario is where the experimental treatment has higher efficacy than the control (alternative 2), particularly for small values of *π*_0_ and *π*_1_. This is probably because the arcsine transformation is designed to stabilise power under the assumption that *π*_0_ = *π*_1_.

##### Summary

Under the assumption that a power-stabilising frontier holds, procedure 4, i.e. ‘conditionally modify margin’ with a threshold *ϵ*= 1.25% on the risk difference scale or *ϵ*= 1.25 on the risk ratio scale, is the best procedure. Power is higher than the ‘do not modify margin’ procedure in almost all scenarios, and type I error is inflated only with the risk difference scale. We next explore two ways to control type I error in this case.

### Controlling type I error rate

#### Smaller fixed *α*

The simplest way of controlling type I error is to widen the confidence intervals using a smaller significance level *α* than the nominal 2.5% (for a one-sided test). We investigate this approach by repeating the base-case simulations for the risk difference, using different significance levels with procedure 4, the smallest threshold for margin modification.

**Fig. 4:**
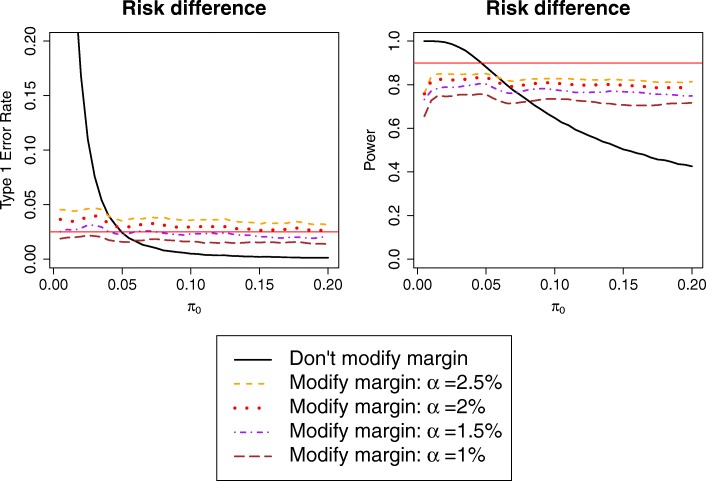
Power and type I error of procedure 4 (‘Conditionally modify margin with small threshold’), with different significance levels. Only presenting the risk difference case, as type I error of the base-case scenario was below the nominal 2.5% level when working on the risk ratio scale

Type I error is always below or around the nominal 2.5% level when using α = 1% (Fig. 4); this leads to a further loss in power of around 8%–9% compared to the ‘do not modify margin’ method. In general, conclusions depend on the relation between expected and observed control event risk:

- *π*_0_ < *π*_*e*0_: the ‘conditionally modify margin’ procedure with α =1% is the only one with type I error within 2.5%, although α =1.5% is close to the nominal level;
- *π*_0_ = *π*_*e*0_: the original sample size calculation was correct, and hence the ‘do not modify margin’ procedure performs well, while the ‘conditionally modify margin’ procedure with smaller α loses ~ 10%–15% power;
- *π*_0_ > *π*_*e*0_: the ‘do not modify margin’ procedure quickly loses power, while all the ‘conditionally modify margin’ procedures are quite stable and have correct type I error for α < 2%.

#### Choose α given control risk

**Fig. 5:**
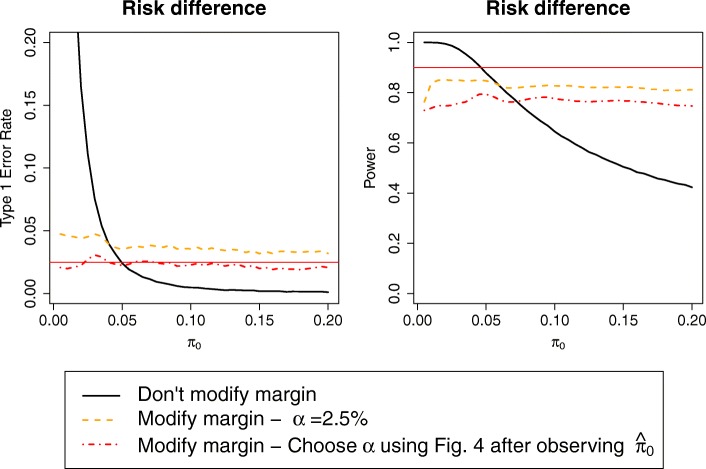
Power and type I error rate of procedure 4 (‘Conditionally modify margin with smallest threshold’), either with standard significance level (one-sided α = 2.5%) or choosing significance level using Fig. 4 after observing control event risk ${\hat{\boldsymbol{\pi}}}_{\mathbf{0}}$ to achieve nominal type I error rate; specifically, in this example we use α = 1% for ${\hat{\boldsymbol{\pi}}}_{\mathbf{0}}<\mathbf{4}\%$ and α = 1.5% otherwise

While one might simply recommend the ‘conditionally modify margin’ procedure with α = 1.5%, this approach may be unnecessarily conservative for control event risks where larger α still leads to good type I error. Hence, another approach could be to choose α after observing the control event risk, using the largest α leading to acceptable type I error for that specific value of the control event risk. This can be estimated from simulations with the desired design parameters analogous to Fig. 4. However, since α is chosen in a data-dependent way, this procedure is not guaranteed to preserve type I error. Nevertheless estimating the type I error from the previous simulations shows the inflation is at most modest (Fig. 5), and hence this approach could be considered acceptable in practice, although it still leads to a 5%–10% loss in power.

A simple way to prevent the additional loss of power is to design the trial using either the smaller fixed α with method i or α at *π*_*e*0_ with method ii.

## Discussion

We have addressed the challenge of designing a non-inferiority trial that preserves power and interpretability of results even when the expected control event risk is badly misjudged. While, statistically, one could argue that sample size re-estimation based on interim analysis, updating the control group event risk and maintaining the original non-inferiority margin solves this problem, in practice substantial increases in sample size are typically not acceptable to funders and may also be challenging for recruitment. Additionally, keeping the margin fixed may not be the optimal choice for the clinical interpretation of results, as demonstrated by the OVIVA trial example. Therefore, alternative statistically principled methods are needed, particularly for the increasing number of non-regulatory trials using non-inferiority designs where previous placebo-controlled trials are often unavailable.

We have proposed methods based on the definition of a non-inferiority frontier. We have argued that a continuously varying frontier might be preferable compared to a fixed risk difference (or risk ratio) frontier to protect against important mis-judgement of the expected control event risk, but that this frontier can be very difficult both to specify and to implement in practice maintaining nominal error rates. We have proposed the power-stabilising frontier as a possible solution, arguing that, on top of its attractive statistical properties, it is often a good compromise between the risk difference and risk ratio frontiers, similar to the ideal clinically determined frontier. Finally, we have proposed and compared three possible ways of implementing such a frontier in the design and analysis of a non-inferiority trial.

This is not the first time that this issue has been tackled in a methodological paper. Recently, Hanscom et al. [20] proposed using baseline or post-randomisation data to re-estimate the non-inferiority margin where this is based on preserving a fraction of the control group effect. Our methods are an alternative that can be prespecified at the trial design stage when there are no clear predictors of control event risk available.

### Extensions

We have considered only binary outcomes, with risk differences and risk ratios as effect measures. Our approach could easily incorporate other effect measures, such as odds ratios or averted infection ratios [23], either to define an alternative non-inferiority frontier, or as the basis of a ‘conditionally modify margin’ procedure assuming the power-stabilising frontier. Similar considerations could be extended to time-to-event outcomes. Again, a non-inferiority frontier could be chosen for absolute differences (e.g. Kaplan–Meier estimates of proportion after a certain time) or relative differences (e.g. hazard ratio).

Non-inferiority trials can have continuous outcomes, for example, the Early Treatment Diabetic Retinopathy Study score (number of letters a patient can read off a chart from a certain distance) in the CLARITY trial [24]. The investigators used an absolute non-inferiority margin of five letters, corresponding to a constant difference non-inferiority frontier. This is appropriate if the margin is independent of the control group mean. Otherwise, if the minimum acceptable number of letters depended on the control group mean, a relative difference, e.g. the ratio of the scores, might be used. However, an important difference compared to binary outcomes is that the sample size (and hence power) calculations for trials with continuous outcomes are independent of the expected control group mean when the variance is not associated with the mean. Hence, power is naturally preserved when assuming a fixed difference frontier.

Future work could investigate how to choose the modification threshold *ϵ* optimally when using the ‘conditionally modify margin’ method.

### Recommendations

Given our results, researchers designing non-inferiority trials with a binary or time-to-event outcome should carefully consider the following:

1. The scale on which the non-inferiority comparison is made should be prespecified in the trial protocol, as it substantially affects trial power (and hence sample size);
2. It is not obvious that the non-inferiority margin should be held fixed (on either risk difference or risk ratio scale) when ${\hat{\pi}}_0$ differs from the expected *π*_*e*0_. Keeping the margin fixed could have implications in terms of power and interpretation, and these need to be considered carefully;
3. A trial design should explicitly prespecify a ‘non-inferiority frontier’, i.e. a curve indicating the tolerable non-inferiority margin for each value of the control event risk. This might be as simple as stating that the non-inferiority margin is fixed on the chosen scale;
4. One possibility is to choose a stepped frontier, but this can be both difficult to define and to implement;
5. Another frontier is based on the arcsine transformation. Although difficult to interpret per se, this is generally an intermediary between the fixed risk difference and fixed risk ratio frontiers, and has the advantage of being the power-stabilising frontier for binomially distributed data. Similar to the stepped frontier, implementation is not straightforward, however;
6. One approach is to test on the arcsine scale and report results on the risk difference scale. However, this generally requires larger sample sizes. Testing on the arcsine scale and reporting on the risk ratio scale is not recommended as it leads to reporting results against large margins or significance levels;
7. An alternative implementation is via our proposed ‘conditionally modify margin’ procedure, which reassesses the margin after observing the control event risk. The trial is still designed and analysed in the usual way, using either a risk difference or a risk ratio margin;
8. When using the ‘conditionally modify margin’ procedure, an appropriate modification threshold can be selected through simulations as here. Functions to perform such simulations are available in the R package dani;
9. If working on the risk difference scale, type I error rate should be controlled using simulations as here to find the appropriate nominal significance level. This has to be done at the design stage of the trial. A conservative approach uses the largest level leading to a rate always below the nominal one, irrespective of the control event risk; otherwise, one can use simulation results to modify the significance level depending on the observed control event risk;
10. The ‘conditionally modify margin’ procedure could potentially be used combined with any other stepped frontier.

## Conclusions

Our proposed method of designing non-inferiority trials through pre-defining a non-inferiority frontier and possibly modifying the non-inferiority margin accordingly after observing the control event risk substantially increases their resilience to inadvertent misjudgements of the control group event risk. The only disadvantage of this method is that, when working on the risk difference scale, some loss of power is expected, and hence sample size should be adjusted accordingly. Explicitly acknowledging before a trial starts that there could be differences between observed and expected control event risks forces researchers to focus in greater depth on the rationale underpinning their choice of non-inferiority margin, and the consequences to the trial if they get these assumptions wrong. While more work is needed to define its use in practice, researchers following our recommendations while designing non-inferiority trials with a binary primary outcome would improve the chance that the trial achieves its aims and will make it resilient to unexpected differences in the control event risk.

## Supplementary information

**Additional file 1.** Additional material include: (i) sample size calculation formulae, (ii) illustrative design and analysis examples and (ii) additional figures of results of the simulations in the Implementation Section.

## Data Availability

All data generated and analysed as part of this study are available at request to the corresponding author.
