# Supplementary material for: Handling an uncertain control group event risk in non-inferiority trials: non-inferiority frontiers and the power-stabilising transformation
Source: Trials. 2020 Feb 6;21:145. doi: 10.1186/s13063-020-4070-4 (PMC7006194; doi:10.1186/s13063-020-4070-4)
Supplement: Supplementary file 1 — Additional file 1. Additional material include: (i) sample size calculation formulae, (ii) illustrative design and analysis examples and (ii) additional figures of results of the simulations in the Implementation Section. [file 13063_2020_4070_MOESM1_ESM.docx]

**Additional material:**

**Appendix A: Sample size calculation formulas**

$n_{0}$= sample size control arm

$n_{1}$= sample size active arm

$\Phi^{-1}$= standard normal quantile function

$\alpha$ = significance level

$\beta$ = type-II error

$\pi_{e0}$ = expected risk in the control arm

$\pi_{e1}$ = expected risk in the active arm

$r=\frac{n_{1}}{n_{0}}$ = allocation ratio

$\delta$ = Non-inferiority margin

**Risk difference:**

$$\boldsymbol{n}_{\boldsymbol{0}}\boldsymbol{=}{\boldsymbol{(\Phi}^{\boldsymbol{-1}}\left( \boldsymbol{1-\alpha} \right)\boldsymbol{+}\boldsymbol{\Phi}^{\boldsymbol{-1}}\left( \boldsymbol{1-\beta} \right)\boldsymbol{)}}^{\boldsymbol{2}}\frac{\boldsymbol{((}\pi_{e0}\boldsymbol{*}\left( \boldsymbol{1-}\pi_{e0} \right)\boldsymbol{+}\pi_{e1}\boldsymbol{*}\left( \boldsymbol{1-}\pi_{e1} \right)\boldsymbol{/r)}}{{\boldsymbol{(}\pi_{e1}\boldsymbol{-}\pi_{e0}\boldsymbol{-}\delta\boldsymbol{)}}^{\boldsymbol{2}}}$$

$$\boldsymbol{n}_{\boldsymbol{1}}\boldsymbol{=}\boldsymbol{r*n}_{\boldsymbol{0}}$$

**Risk ratio:**

$\boldsymbol{n}_{\boldsymbol{0}}\boldsymbol{=}{\boldsymbol{(\Phi}^{\boldsymbol{-1}}\left( \boldsymbol{1-\alpha} \right)\boldsymbol{+}\boldsymbol{\Phi}^{\boldsymbol{-1}}\left( \boldsymbol{1-\beta} \right)\boldsymbol{)}}^{\boldsymbol{2}}\frac{\boldsymbol{((}\left( \boldsymbol{1-}\pi_{e0} \right)\boldsymbol{/(}\pi_{e0}\boldsymbol{)+}\left( \boldsymbol{1-}\pi_{e1} \right)\boldsymbol{/(r*}\pi_{e1}\boldsymbol{))}}{{\boldsymbol{(log(}\pi_{e1}\boldsymbol{/}\pi_{e0}\boldsymbol{)-}\delta\boldsymbol{)}}^{\boldsymbol{2}}}$

$$\boldsymbol{n}_{\boldsymbol{1}}\boldsymbol{=r*}\boldsymbol{n}_{\boldsymbol{0}}$$

**Arc-sine difference:**

$$\boldsymbol{n}_{\boldsymbol{0}}\boldsymbol{=}{\boldsymbol{(\Phi}^{\boldsymbol{-1}}\left( \boldsymbol{1-\alpha} \right)\boldsymbol{+}\boldsymbol{\Phi}^{\boldsymbol{-1}}\left( \boldsymbol{1-\beta} \right)\boldsymbol{)}}^{\boldsymbol{2}}\frac{\boldsymbol{(}\frac{\boldsymbol{1}}{\boldsymbol{4}}\boldsymbol{+}\frac{\boldsymbol{1}}{\boldsymbol{4}\boldsymbol{r}}\boldsymbol{)}}{{\boldsymbol{(asin(}\sqrt{\pi_{e1}}\boldsymbol{)-asin(}\sqrt{\pi_{e0}}\boldsymbol{)-}\delta\boldsymbol{)}}^{\boldsymbol{2}}}$$

$$\boldsymbol{n}_{\boldsymbol{1}}\boldsymbol{=}\boldsymbol{r*n}_{\boldsymbol{0}}$$

# Appendix B: Illustrative design and analysis examples

In this appendix we provide examples of the design and analysis of hypothetical trials following the methods presented in this paper. We compare the results with those from the analysis of a trial assuming either a fixed risk difference or risk ratio frontier. For all the examples, design parameters are as in the base-case scenario of our simulation study, i.e. $\pi_{e0}=\pi_{e1}=5\%$ , $\pi_{f1}=10\%$ , power = 90%, one-sided $\alpha=2.5\%$ and r = 1.

***Design***

As described in the main text, because of the different shapes of the non-inferiority frontier, with these same design parameters, the estimated total sample sizes for standard non-inferiority trials designed with a fixed risk difference, fixed risk ratio, and fixed arc-sine difference are 800, 1,664 and 1,136 respectively.

To incorporate resilience to unanticipated variation in the control event risk into a design using the fixed risk difference scale by conditionally modifying the risk difference margin with a threshold $\epsilon=$ 1.25% requires either a data-dependent choice of one-sided alpha at the end of the trial or a more conservative upfront lowering of the one-sided alpha. As α=1.5% is the acceptable significance level when the expected and observed control event risk match (Figure 4 main text), sample size is inflated to 903 using the first method (13% increase from the standard sample size calculation on the risk difference scale α=2.5%), while for the second method this goes up to 990 (23% increase), both still smaller than designing on the arc sine scale (1,136). However, as the margin is wrongly modified ~20% of the times using the smallest threshold ε, actual power may be slightly lower than the nominal level. Future work will explore how to perform a more precise sample size calculation.

***Analysis***

For simplicity, we use the same dataset for all analysis methods; although some designs require different sample sizes as above, here we illustrate the methods using the sample size required to reach 90% power to prove non-inferiority on the arc-sine difference scale within$\mathrm{asin} \left( \sqrt{\pi_{f1}} \right)- \mathrm{asin} \left( \sqrt{\pi_{e0}} \right)= 0.096$, , i.e. 568 patients per arm, total 1,136 patients.

We show how to analyse a trial with each of the following methods:

- Test and report on the arc-sine scale, as in Section 3.1;

- Test on the arc-sine scale, report on the risk difference scale changing the margin, as in Section 3.2(i);

- Test on the arc-sine scale, report on the risk difference scale changing significance level and modifying the margin, as in Section 3.2(ii);

- Test and report on the risk difference scale, as per a standard non-inferiority trial;

- Test on the risk difference scale, modifying the margin if $\left| \hat{\pi}_{0}-\pi_{e0} \right|>1.25\%$ and testing with $\alpha=1\%$, as in Section 3.5(i).

- Test on the risk difference scale, modifying the margin if $\left| \hat{\pi}_{0}-\pi_{e0} \right|>1.25\%$ and choose $\alpha$ using Figure (4), as in Section 3.5(ii).

- Test and report on the risk ratio scale, as per a standard non-inferiority trial on this scale;

- Test on the risk ratio scale, modifying the margin if $\left| log(\hat{\pi}_{0}/\pi_{e0}) \right|>log(1.25)$ and testing with $\alpha=2.5\%$, as in Section 3.4.

*(i) Example 1: actual control and intervention event risks are higher than anticipated*

First we consider the analysis of a trial where the observed event risks in the control and active arms are 5% higher than $\pi_{e0}$ and $\pi_{e1}$ respectively, i.e. $\hat{\pi}_{0}=10\% , \hat{\pi}_{1}=10\%.$ In our hypothetical example trial, we observe 57 events in both the control$(\hat{\pi}_{0}=10\%)$ and active arm $(\hat{\pi}_{1}=10\%).$

**Test and report on the arc-sine scale.** The estimated arc-sine difference and associated standard error are:

$$\hat{AS}=\mathrm{asin} \left( \sqrt{\hat{\pi}_{1}} \right)- \mathrm{asin} \left( \sqrt{\hat{\pi}_{0}} \right)= 0.000 \hat{\sigma}_{AS}=\sqrt{\frac{1}{4n_{0}}+\frac{1}{4n_{1}}}=0.030$$

Hence, the Z statistic is:

$$Z_{AS}= \frac{\hat{AS}-\delta_{AS}}{\hat{\sigma}_{AS}}= -3.244$$

So, the p-value for the test of non-inferiority within margin 0.096 gives a p-value = $\Phi^{-1}\left( Z_{AS} \right)<0.01$, providing evidence that the new treatment is non-inferior. The two-sided 95% confidence interval for the arc-sine difference is: [-0.058; 0.058].

**Test on the arc-sine scale, report on the risk difference scale changing the margin.** Since the test is performed on the arc-sine scale, and the trial has been designed on this scale, the Z statistic and p-value are the same as above. The only difference is that we report the results on the risk difference scale by calculating the non-inferiority margin leading to the same Z statistic. Following Section 3.2(i), we first estimate the risk difference and its associated standard error:

$$\hat{RD}= \hat{\pi}_{1}-\hat{\pi}_{0}=0.0\% \hat{\sigma}_{RD}=\sqrt{\frac{\hat{\pi}_{0}\left( 1-\hat{\pi}_{0} \right)}{n_{0}}+\frac{\hat{\pi}_{1}\left( 1-\hat{\pi}_{1} \right)}{n_{1}}}=1.8\%$$

And then we find for which non-inferiority margin $\delta_{RD}$ these lead to the same Z statistic as $Z_{AS}$:

$$\delta_{RD}=\hat{RD}-Z_{AS}*\hat{\sigma}_{RD}= 5.8\%$$

Hence, we report that we found the new treatment was non-inferior within the 5.8% risk difference margin, with p<0.01 and two-sided 95% confidence interval [-3.5%; 3.5%].

**Test on the arc-sine scale, report on the risk difference scale changing significance level.** Here, the estimated risk difference and associated standard error are the same as for the previous method. However, the non-inferiority margin is back-calculated from the power-stabilising frontier as:

$$\delta_{RD}^{*}={sin(asin(\sqrt{\hat{\pi}_{0}})+asin(\sqrt{\pi_{f1}})-asin(\sqrt{\pi_{e0}}))}^{2}-\hat{\pi}_{0}=6.5\%$$

The Z statistic with this larger non-inferiority margin is:

$$Z_{RD}=\frac{\hat{RD}-\delta_{RD}^{*}}{\hat{\sigma}_{RD}}=--3.639$$

Hence, the ratio between $Z_{RD}$ and $Z_{AS}$ is equal to 1.12, and $z_{1-\alpha^{*}}=1.11z_{1-\alpha}=2.20.$ This is true for one-sided $\alpha^{*}=1.4\%$.

In conclusion, we proved non-inferiority within the 6.5% risk difference margin at the 1.5% one-sided significance level (p<0.01), with 97.2% two-sided confidence interval [-3.9%; 3.9%].

**Test and report on risk difference scale.** This is a standard non-inferiority trial assuming a fixed risk difference frontier. Although an improved method would be preferable here to perform the test due to better efficiency^1^, we retain the simple test using normal theory, for simplicity and comparability with the other methods. The estimated risk difference and associated standard error are the same as with the previous methods, i.e. $\hat{RD}=0.0\%$ and $\hat{\sigma}_{RD}=1.8\%$. However, here we test at the pre-defined 5% non-inferiority margin:

$$Z_{AS}= \frac{\hat{RD}-\delta_{RD}}{\hat{\sigma}_{RD}}= -2.778 p<0.01$$

Finally, we report the 95% confidence interval for the risk difference, i.e. [-3.5%; 3.5%], and conclude that we provided evidence of non-inferiority at the 5% risk difference margin (p=0.50).

**Test on the risk difference scale with α=1%, modifying the margin.** Here, we start again from the same estimates of risk difference and associated standard error:

$$\hat{RD}=0.0\% \hat{\sigma}_{RD}=1.8\%$$

In this example, since $\left| \hat{\pi}_{0}-\pi_{e0} \right|=5\%>1.25\%$, we modify the margin from the initially intended 5% to the one back calculated from the arc-sine frontier given $\hat{\pi}_{0}$, i.e. 6.5%. Hence, the Z statistic and p-value are:

$$Z_{RD}= \frac{\hat{RD}-\delta_{RD}^{*}}{\hat{\sigma}_{RD}}= -3.639 p<0.01$$

In conclusion, we prove non-inferiority at one-sided 2.5% significance level within the 6.5% margin (p<0.01), and the two-sided 95% confidence interval for the risk difference is [-4.2%; 4.2%], larger than with the fixed risk difference example as we have used α=1% in order to control type 1 error.

**Test on the risk difference scale, modifying the margin and choosing α using Figure 4**. This method is similar to the previous one, but this time the significance level for testing is chosen from Figure 4, given that $\hat{\pi}_{0}=10\%$. In this specific case, this leads to using a one-sided α=1.5%, and hence the confidence interval is now [-3.9%; 3.9%].

**Test and report on the risk ratio scale.** The estimated log-risk ratio and associated confidence interval are:

$$\hat{RR}=log(\hat{\pi}_{1})-log(\hat{\pi}_{0})=0.00 \hat{\sigma}_{RR}=\sqrt{\frac{\left( 1-\hat{\pi}_{0} \right)}{n_{0}\hat{\pi}_{0}}+\frac{\left( 1-\hat{\pi}_{1} \right)}{n_{1}\hat{\pi}_{1}}}=0.18$$

The non-inferiority margin on the log-risk ratio scale is $\delta_{RR}=log \left( \frac{0.1}{0.05} \right)=0.69$, leading to the following Z statistic for the test on the log-risk ratio scale:

$$Z_{RR}= \frac{\hat{RR}-\delta_{RR}}{\hat{\sigma}_{RR}}= -4.29 p<0.01$$

There is therefore strong evidence at the one-sided 2.5% significance level that the new treatment is non-inferior to the control within a log-risk ratio margin of 0.69, i.e. that the relative risk is less than 2. Note that since we are working with a fixed risk ratio scale, and $\hat{\pi}_{0}=10\%$, we would now be happy to tolerate up to $\hat{\pi}_{1}=20\%$. In this case the 95% confidence interval should be reported on the risk ratio scale: [0.71, 1.42].

**Test and report on the risk ratio scale, modifying the margin.** Since $\left| log(\hat{\pi}_{0}/\pi_{e0}) \right|=log(2)>log(1.25)$, the margin is modified according to the power-stabilising frontier:

$$\delta_{RR}^{*}={log(sin(asin(\sqrt{\hat{\pi}_{0}})+asin(\sqrt{\pi_{f1}})-asin(\sqrt{\pi_{e0}}))}^{2})-log(\hat{\pi}_{0})=0.50$$

The estimated log-risk ratio and standard error are as before:

$$\hat{RR}=0.00 \hat{\sigma}_{RR}=0.18$$

Differently from the risk difference case, in this situation there is no need to adjust for type-1 error rate, and hence we can keep one-sided α=2.5% as significance level. The Z statistic and p-value are:

$$Z_{RR}= \frac{\hat{RR}-\delta_{RR}^{*}}{\hat{\sigma}_{RR}}= -3.11 p<0.01$$

The 95% confidence interval on the risk ratio scale is [0.71; 1.42], and the p-value for the non-inferiority test within the $e^{0.5}=1.65$ margin is < 0.01.

*(ii) Other example*s**.** Table (a) below shows the results of the analysis performed on the previous example and on two additional examples. These are all based on the same design parameters, i.e. those for the base-case scenario of our simulation study, leading to a sample size of 568 patients per arm. However, in the second example $\hat{\pi}_{1}=15\%$, so that the observed active and control event risk differ by 5%, while in the third example $\hat{\pi}_{0}=6\% , \hat{\pi}_{1}=10\%$, so that the active event risk is close to its expected value at the design stage, and hence we do not modify the margin when using methods from Section 3.4 and 3.5.

| Method | Two-sided CI Level | CI | Margin | 1-sided α level | p |
| --- | --- | --- | --- | --- | --- |
| Example 1:  $\hat{\pi}_{0}=10\% , \hat{\pi}_{1}=10$% |  |  |  |  |  |
| Test & report on arc-sine scale | 95% | [-0.058; 0.058] | 0.096 | 2.5% | <0.01 |
| Test arc-sine, report RD (change margin) | 95% | [-3.5%; 3.5%] | 5.7% | 2.5% | <0.01 |
| Test arc-sine, report RD (change α) | 97.2% | [-3.9%; 3.9%] | 6.5% | 1.4% | <0.01 |
| Test RD | 95% | [-3.5%; 3.5%] | 5% | 2.5% | <0.01 |
| Test RD, Modify Margin, α=1% | 95% | [-4.2%; 4.2%] | 6.5% | 1% | <0.01 |
| Test RD, Modify Margin, choose α | 95% | [-3.9%; 3.9%] | 6.5% | 1.5% | <0.01 |
| Test RR | 95% | [0.71, 1.42] | 2 | 2.5% | <0.01 |
| Test RR, Modify Margin | 95% | [0.71, 1.42] | 1.65 | 2.5% | <0.01 |
| Example 2:  $\hat{\pi}_{0}=10\% , \hat{\pi}_{1}=15$% |  |  |  |  |  |
| Test & report on arc-sine scale | 95% | [0.018; 0.134] | 0.096 | 2.5% | 0.25 |
| Test arc-sine, report RD (change margin) | 95% | [1.2%; 8.8%] | 6.3% | 2.5% | 0.25 |
| Test arc-sine, report RD (change α) | 97.0% | [0.8%; 9.2%] | 6.5% | 1.5% | 0.25 |
| Test RD | 95% | [1.2%; 8.8%] | 5% | 2.5% | 0.50 |
| Test RD, Modify Margin, α=1% | 95% | [0.3%; 9.7%] | 6.5% | 1% | 0.25 |
| Test RD, Modify Margin, choose α | 95% | [0.8%; 9.2%] | 6.5% | 1.5% | 0.25 |
| Test RR | 95% | [1.10, 2.06] | 2 | 2.5% | 0.04 |
| Test RR, Modify Margin | 95% | [1.10; 2.06] | 1.65 | 2.5% | 0.29 |
| Example 3:  $\hat{\pi}_{0}=6\% , \hat{\pi}_{1}=10$% |  |  |  |  |  |
| Test & report on arc-sine scale | 95% | [0.016; 0.132] | 0.096 | 2.5% | 0.23 |
| Test arc-sine, report RD (change margin) | 95% | [0.9%; 7.2%] | 5.2% | 2.5% | 0.23 |
| Test arc-sine, report RD (change α) | 97.4% | [0.4%; 7.6%] | 5.4% | 1.3% | 0.23 |
| Test RD | 95% | [0.9%; 7.2%] | 5% | 2.5% | 0.27 |
| Test RD, Modify Margin, α=1% | 95% | [0.2%; 7.7%] | 5% | 1% | 0.27 |
| Test RD, Modify Margin, choose α | 95% | [0.5%; 7.5%] | 5% | 1.5% | 0.27 |
| Test RR | 95% | [1.11, 2.51] | 2 | 2.5% | 0.20 |
| Test RR, Modify Margin | 95% | [1.11; 2.51] | 2 | 2.5% | 0.20 |

Table (a): Results of analysis of hypothetical trials using methods presented in this paper and standard non-inferiority designs on either the risk difference or risk ratio scales. For each method and example, we provide confidence level and corresponding interval, Non-inferiority margin, 1-sided significance level for testing and p-value. When using the “Modify Margin” methods, the 1-sided significance level used for testing can be modified without this affecting confidence level; this is because a different α is used only to maintain type-1 error below the nominal 2.5%, and hence the relevant confidence level is still two-sided 95%.

**Bibliography:**

1. Newcombe RG. Interval estimation for the difference between independent proportions: comparison of eleven methods. *Stat Med* 1998; 17: 873–890.

Figure (a): total sample size (2 groups) to achieve 90% power for varying control event risks using non-inferiority margins defined on the risk difference (left panel) and risk ratio (right) scales (two-sided alpha=0.05).

Figure (b): power for given sample size for varying control event risks using non-inferiority margins defined on the risk difference (N=400, left panel) and risk ratio (N=832, right panel) scales (two-sided alpha=0.05)

Figure (c): Proportion of margins modified using the three different "Conditionally modify margin" procedures. Data are generated according to the base-case scenario of Table 1 for testing type I error rate.

Figure (d): Type I error (top) and power (bottom) of procedure 3 ("Conditionally modify margin with medium threshold"), using the risk difference (left) or risk ratio (right) scale. Data are generated according to the alternative scenarios of Table 1 for varying values of control event risk.

Figure (e): Type I error (top) and power (bottom) of procedure 2 ("Conditionally modify margin with largest threshold"), using the risk difference (left) or risk ratio (right) scale. Data are generated according to the alternative scenarios of Table 1 for varying values of control event risk.
